# How Coaches Can Improve Their Teams’ Match Performance—The Influence of In-Game Changes of Tactical Formation in Professional Soccer

**DOI:** 10.3389/fpsyg.2022.914915

**Published:** 2022-06-09

**Authors:** Leon Forcher, Leander Forcher, Darko Jekauc, Hagen Wäsche, Alexander Woll, Timo Gross, Stefan Altmann

**Affiliations:** ^1^Institute of Sports and Sports Science, Karlsruhe Institute of Technology, Karlsruhe, Germany; ^2^TSG 1899 Hoffenheim, Zuzenhausen, Germany; ^3^TSG ResearchLab gGmbH, Zuzenhausen, Germany

**Keywords:** football, tactics, game analysis, video, scouting, trainer

## Abstract

The tactical formation has been shown to influence the match performance of professional soccer players. This study aimed to examine the effects of in-game changes in tactical formation on match performance and to analyze coach-specific differences. We investigated three consecutive seasons of an elite team in the German Bundesliga which were managed by three different coaches, respectively. For every season, the formation changes that occurred during games were recorded. The match performance was measured on a team level using the variables “goals,” “chances,” and “scoring zone” entries (≙successful attacking sequence) for the own/opposing team. Non-parametric tests were used to compare the 10 min before with the 10 min after the formation change, as well as games with and without formation change. In the 10 min after the formation change, the team achieved more goals/chances/scoring zone entries than in the 10 min before the formation change (mean ES = 0.52). Similarly, the team conceded fewer opposing goals/chances/scoring zone entries in the 10 min after the formation change (mean ES = 0.35). Furthermore, the results indicate that the success of the respective formation change was dependent on the responsible coach. Depending on the season, the extent of the impacts varied (season 1: mean ES = 0.71; season 2: mean ES = 0.26; and season 3: mean ES = 0.22). Over all three seasons, the formation changes had a positive effect on the match performance of the analyzed team, highlighting their importance in professional soccer. Depending on the season, formation changes had varying impacts on the performance, indicating coach-specific differences. Therefore, the quality of the formation changes of the different coaches varied. The provided information can support coaches in understanding the effects of their in-game decisions.

## Introduction

In recent years, scientific interest in soccer match performance has markedly increased. Physical and technical match performance has been investigated frequently (Dolci et al., 2020; Forcher et al., 2022b). Furthermore, since computer technology and science allowed researchers to deal with larger data sets, the construct of the tactical soccer performance received increasing attention (Sarmento et al., 2018). Particularly, current reviews highlight the offensive and defensive tactical performance of single players, groups, and whole teams, thus pointing to the great opportunities in-game analysis research (Lepschy et al., 2018; Goes et al., 2020; Forcher et al., 2022a). Similarly, the interest in the influence of tactical factors on soccer performance has also increased recently (Modric et al., 2020; Vilamitjana et al., 2021).

Typical tactical factors that influence the match performance of soccer players are the playing position or the tactical formation. It is widely accepted that the playing position has a large impact on technical as well as physical match performance (Dolci et al., 2020). For example, central midfielders indicate more ball-possessions than other positions (Dellal et al., 2010) and wide positions (defenders & midfielders) run the greatest distances at high-intensity and sprinting speed zones (Rivilla-Garcia et al., 2018; Aquino et al., 2020; Paraskevas et al., 2020). Similarly, the tactical formation of a soccer team impacts the match performance of a single player and the whole team. Teams playing in a formation with three central defenders (e.g., 3-5-2) tend to be more physically demanded in comparison to teams with two central defenders (e.g., 4-4-2; Forcher et al., 2022b). By contrast, looking at the technical performance, players in a 4-4-2 formation display more passes than in other formations (Bradley et al., 2011; Arjol-Serrano et al., 2021). Lastly from a tactical perspective, teams in a 3-5-2 formation can be more compact and, therefore, can put more pressure on the opposing attacking team than teams in a 4-4-2 formation (Memmert et al., 2019). To summarize, both tactical factors (i.e., playing position and tactical formation) have an influence on soccer match performance.

Nevertheless, the studies that examined the effects of tactical formation on match performance have some distinctive features. Specifically, all the mentioned studies that investigated tactical formations focused on the effects of tactical formation changes that occurred *between* two or more games. Besides substitutions, such changes in tactical formation within a game are one way for the coach to potentially influence the running of the game (Bradley et al., 2014). To the best of the authors’ knowledge, until today, no studies analyzed the effects of changing the tactical formation *within* one game.

Apart from this, the studies mentioned above have common features that differ from the approach taken in this study. The majority of studies investigated physical and technical parameters (Bradley et al., 2011; Rivilla-Garcia et al., 2018; Paraskevas et al., 2020) to describe soccer performance. Incidentally, most of the previous studies focused on individual match performance metrics and have not studied parameters that are directly linked to success. In contrast, the parameters investigated in the current study are linked in a more direct way to success (Lepschy et al., 2020). In addition, most of the investigations dealt with single players’ game performances. As suggested in previous studies, we divided the game into individual attacking sequences (Forcher et al., 2021). Subsequently, we assessed the success of each individual ball possession for the own as well as for the opposing team.

In conclusion, it seems worthwhile to investigate the effects of in-game formation changes using outcome variables that are linked to success in soccer such as goals, chances, and last-plane entries. Accordingly, the current study aimed to examine the effects of such in-game changes in the tactical formation on match performance by analyzing both the own team’s and opposing team’s attacking sequences. In addition, we sought to identify possible coach-specific differences regarding these effects. The results of our study could help to detect the impact of in-game formation changes and evaluate coach-specific differences on these dynamics.

## Materials and Methods

### Study Design

In the present study, three consecutive seasons of a German Bundesliga team were analyzed (season 1 = 2021/22; season 2 = 2019/20; and season 3 = 2018/19). To detect changes in tactical formation that occurred within games [in-game], we analyzed each game by observation. To quantify if and to which extent the in-game formation change influenced the match performance, we conducted two comparisons. First, we analyzed the effects of in-game formation changes by comparing games with at least one formation change in contrast to games without a formation change. Second, we analyzed the in-game effects of formation changes by comparing the 10 min before [10 min pre] to the 10 min after [10 min post] the formation change. The 10-minute period represents a compromise between an acceptable number of attacking sequences and an exclusion of other impacts.

In order to quantify the effects of the in-game formation changes on an attacking sequence level, goals, chances, and scoring zone entries were analyzed for the own as well as for the opposition team, leading to a total of six different variables.

### Sample

In this study, official video data of three consecutive seasons of a German elite team in the Bundesliga were analyzed, which were provided by Wyscout (Wyscout, Chiavari, Italy). During this period, the club participated in international competitions (UEFA Champions League & UEFA Europa League) in two of the three seasons and was managed by three different coaches. In the second season, the coach was replaced after the 30th matchday and, therefore, only 30 of 34 possible games of this season were analyzed. The other two seasons consisted of 34 games each. Accordingly, the sample comprised a total of 98 games. Since each season was trained by a different coach, differences between the seasons may be due to differences between the coaches. The study was conducted according to the guidelines of the Declaration of Helsinki and approved by the local ethics committee (Human and Business Sciences Institute, Saarland University, Germany, identification number: 22–02, 10 January 2022).

### Procedures

The tactical formation was defined as the distribution of the players on the pitch and was only observed in controlled build-up play from either their own or opposing team. Defensive (opposing team in ball possession) and offensive (own team in ball possession) tactical formations were distinguished. A tactical formation is defined by the number of players that play as defenders, midfielders, and forwards (i.e., 4-4-2: 4 defenders, 4 midfielders, and 2 forwards). Two experienced video analysts independently recorded every formation change by observation and when differences arose they were discussed until a consensus was reached.

A formation change was recorded if the analyzed team either changed solely their offensive formation, changed solely their defensive formation, or changed both formations simultaneously. A change in the tactical formation (e.g., number of players per playing position: i.e., defenders, midfielders, and forwards) was counted when the new tactical formation was maintained throughout a minimum of two consecutive build-up play phases. The defensive formation was monitored when the opposing team was in ball possession, whereas the offensive formation was monitored when the own team was in ball possession. The opposing teams’ tactical formation was not considered in this study. Afterward, the exact time point for every single formation change was identified. The time point was defined as the first build-up play phase in which the change of the tactical formation was observed.

To detect the effects of in-game formation changes on goals, chances, and scoring zone entries, we analyzed *games with at least one formation change* in comparison with *games without a formation change*. In addition, the 10 min before the formation change [*10 min pre*] were compared to the 10 min afterward [*10 min post*]. The 10-minute period was chosen because it represents a compromise between an acceptable number of attacking sequences and an exclusion of possible impacts by an opposing adaption to the formation change.

The match performance on a team level was analyzed using six different key performance variables that assess the success of individual attacking sequences. For the own teams as well as for the opposition team, goals, chances, and scoring zone entries were recorded. As the main goal of an attacking sequence is to score, goals and chances were recorded in order to quantify the success of an individual attacking sequence (Tenga et al., 2010; González-Rodenas et al., 2019). Additionally, by recording scoring zone entries, a further key performance variable was considered. Scoring zone entries are an expressive variable when looking at the match performance of a whole team and evaluating the success of an individual attacking sequence (Guimarães et al., 2021). Every goal and every chance arise after a scoring zone entry.

Similar to Tenga et al. (2010), we defined chances as every shot or header that was executed in the penalty area. Additionally, every shot from outside the penalty area that led to a goalkeeper save was counted as a *chance*.

**Figure 1:**
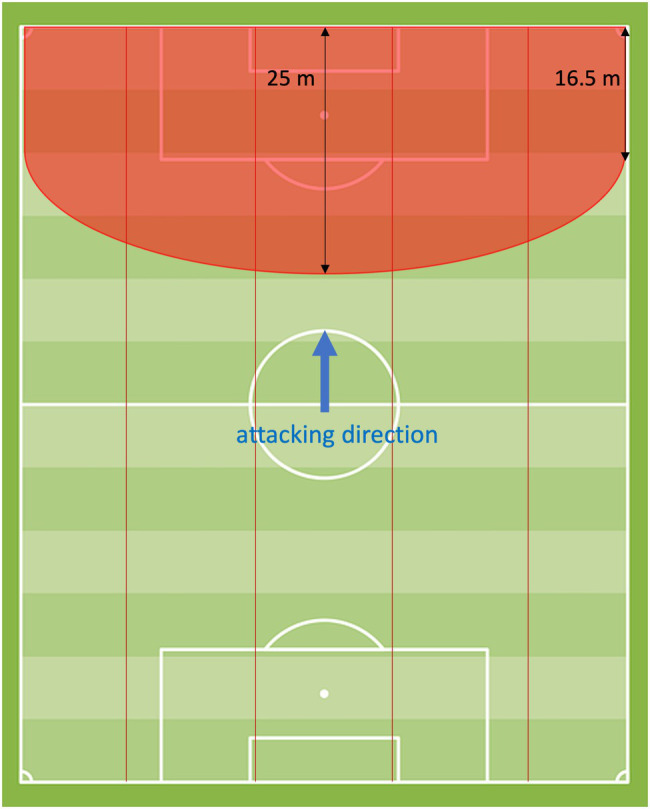
Scoring zone.

The scoring zone is a zone on the pitch that spreads in front of the opposition goal (Figure 1). The area starts at the goal-line up to the corners and continues with a semicircle from side-line to side-line. Since Guimarães et al. (2021) revealed that attacks *via* the central zone of the final third are more promising than attacks *via* the outside lanes, the *scoring zone* area is larger in the center than on the outside. Therefore, on the side-lines, the semicircle originates with a horizontal distance of 16.5 m to the goal-line. At its most distant point, the center of the goal-line, the distance between the semicircle and the goal-line constitutes 25 m. A scoring zone entry was counted if a player of the attacking team has a ball contact in the scoring zone area and is facing toward the goal. Further, a scoring zone entry was counted if the player in ball possession faced the opposing goal even if he was not in the scoring zone area and a maximum of six players of the defending team were in front of the ball. Therefore, in addition to chances and goals, scoring zone entries were considered as a successful attacking sequence.

**Figure 2:**
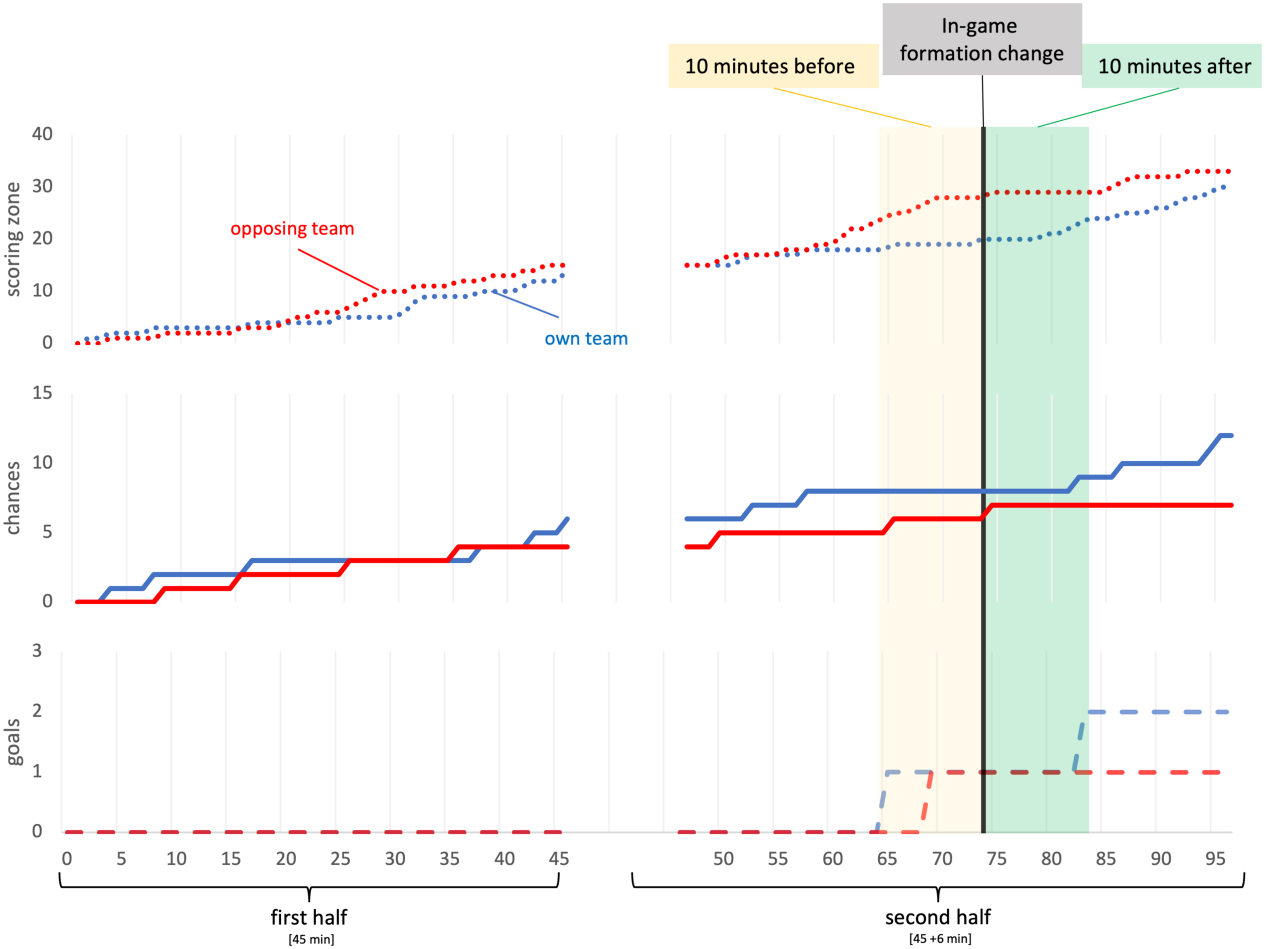
Example.

In Figure 2, a visual presentation of one game is provided. The six different variables were listed throughout every minute of the whole game time. Further, the moment of the in-game formation change of the own team was tagged and the 10 min-pre and 10 min-post-phases were outlined.

Moreover, to investigate the inter-rater reliability of the key performance variables studied (goals, chances, and scoring zone), a game from the first season was evaluated by two experienced analysts (see Supplementary Table S1). Given the high agreement between the results of both analysts (mean Cohen’s Kappa = 0.94; mean *p* = 0.02), the applied procedure can be considered reliable (Landis and Koch, 1977).

### Statistical Analysis

To detect the impact of in-game formation changes, mean values and standard deviations [SD] for goals, chances, and scoring zone entries were calculated for *games with at least one formation change* and *games without formation change*. In addition, for all games with at least one formation change, these variables were examined *10-min-pre formation change* and *10-min-post formation change*.

All variables were checked for normal distribution with the help of Kolmogorov–Smirnov tests. Since not all variables were normally distributed, we performed the statistical analysis with non-parametric tests (see Supplementary Table S2).

Moreover, to evaluate the differences between the three coaches, we considered each season separately.

First, we compared games with and games without formation change. The number of games was not equally distributed throughout the two groups (i.e., games with and without formation change).

Therefore, to detect possible differences between the games with and without formation change, Mann–Whitney-U-tests were conducted.

Second, data from 10-min-pre formation change were compared to 10-min-post formation change. Specifically, for each formation change detected, data were collected for the 10-min pre- and 10-min post-phases so that paired samples were provided. Therefore, to determine whether the measured variables increase or decrease in the 10-min-post phase compared to the 10-min-pre phase, sign-tests were executed.

To determine the magnitude of the group differences, Cohen’s d effect sizes [ES] were calculated for every group comparison. In detail, small (0.2 ≤ ES < 0.5), medium (0.5 ≤ ES < 0.8), and large (ES ≥ 0.8) ES were distinguished (Cohen, 1988).

All statistical analyses were executed using IBM SPSS Statistics 25.0.0.0 (IBM Co., New York, USA). Due to the expected low number of formation changes per season, we mainly referred to effect sizes when interpreting the results instead of *p* values.

## Results

**Figure 3:**
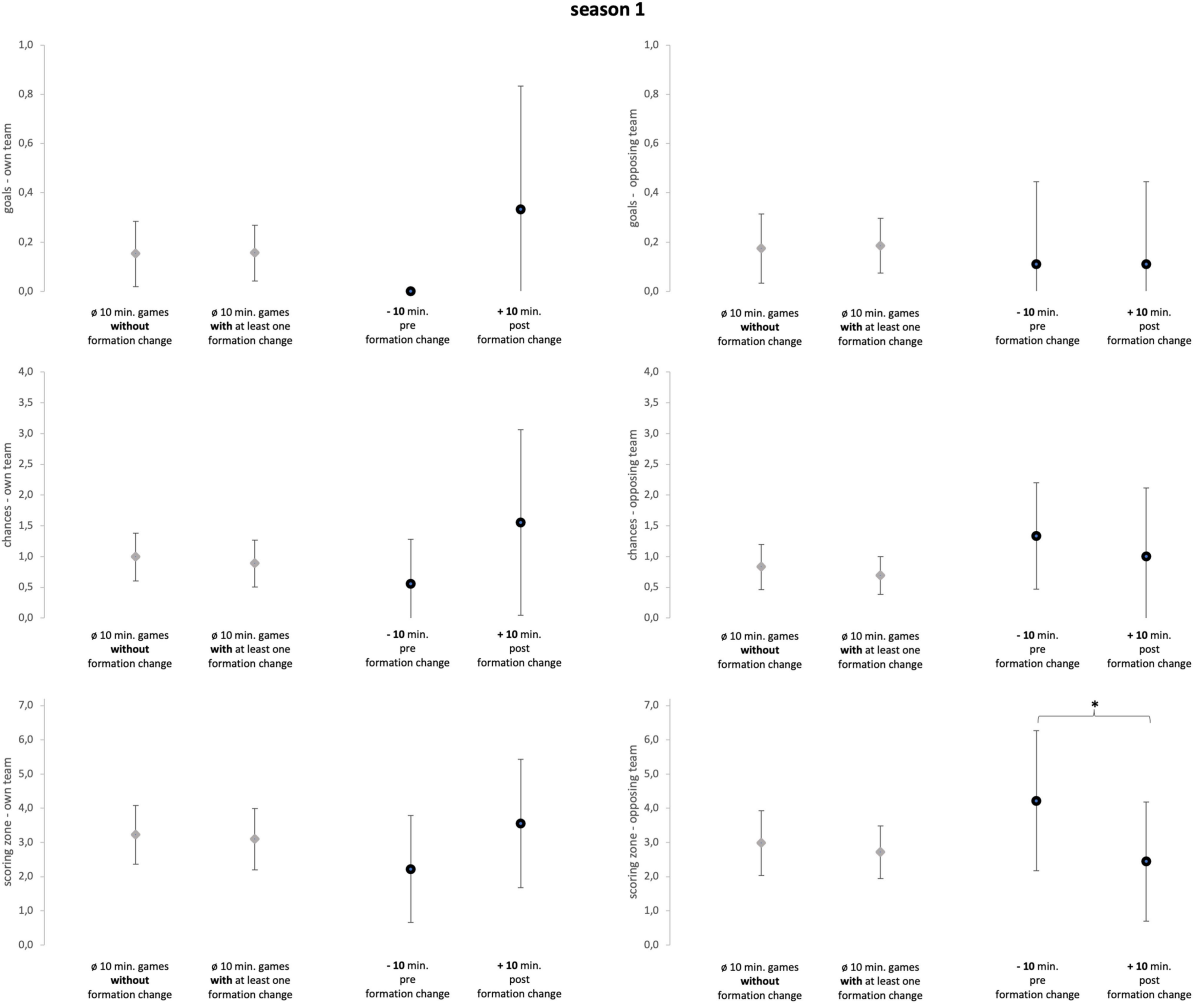
Season 1. Data of season 1 are presented as mean values ± SD. Black parentheses indicate significant differences (*p* < 0.05).

Season 1 (Figure 3) included nine games with a formation change, resulting in nine single formation changes that were investigated. Of the nine changes, eight were recorded in the second half leading to an average game minute of 64.11(±15.57). Seven changes concerned both offensive and defensive formation, while only one change concerned solely defensive or offensive formation, respectively.

**Figure 4:**
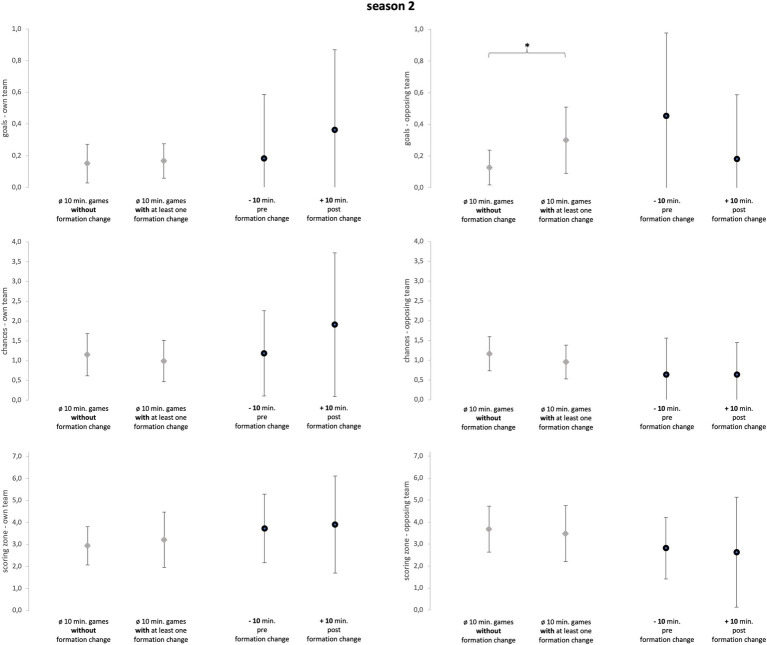
Season 2. Data of season 2 are presented as mean values ± SD. Black parentheses indicate significant differences (*p* < 0.05).

Season 2 (Figure 4) included 10 games with a formation change, resulting in 11 single formation changes that were investigated (one game with two formation changes). All 11 changes were recorded in the second half leading to an average game minute of 55.82 (±13.20). Five changes concerned both offensive and defensive formation, two changes only defensive formation, and four changes only offensive formation.

**Figure 5:**
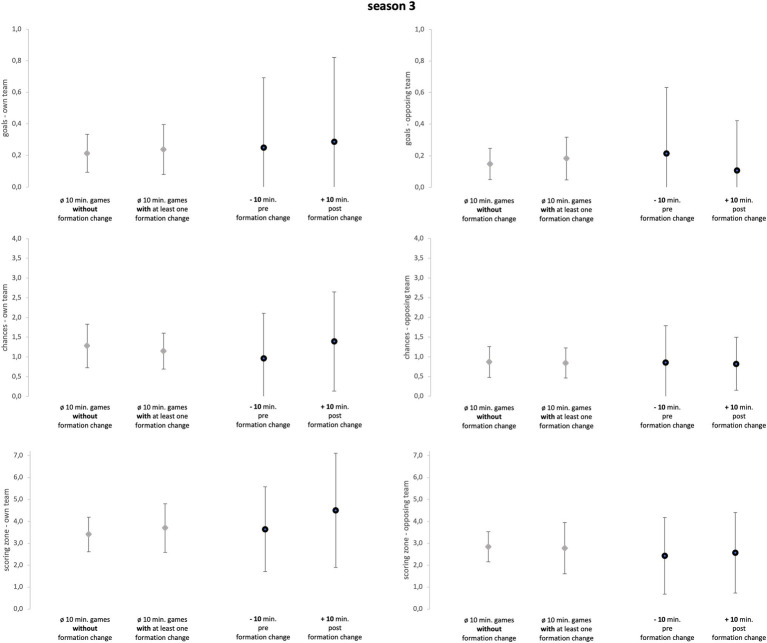
Season 3. Data of season 3 are presented as mean values ± SD. Black parentheses indicate significant differences (*p* < 0.05).

Season 3 (Figure 5) included 22 games with a formation change, resulting in 28 single formation changes that were investigated (six games with two formation changes). A 23 of the 28 changes were recorded in the second half leading to an average game minute of 55.46(±17.45). A 16 changes concerned both offensive and defensive formation, eight changes only defensive formation, and four changes only offensive formation.

**Figure 6:**
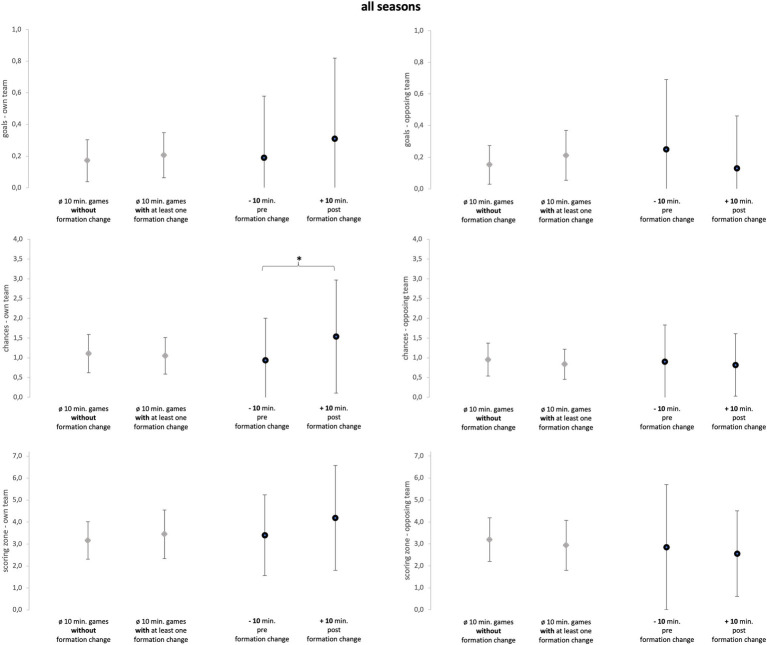
All seasons. Data of all season are presented as mean values ± SD. Black parentheses indicate significant differences (*p* < 0.05).

Descriptive statistics (mean ± SD) of every season separately and all seasons taken together and for every variable (goals, chances, and scoring zone) are reported in Figures 3–6. Numerical values can be taken from the Supplementary Tables S3, S4. Detailed information on each in-game formation change including defensive and offensive formations before and after the change can be found in Supplementary Table S6.

The Mann–Whitney-U-tests (see Figures 3–6), comparing games with formation change and without formation change, revealed that the analyzed team in season 2 conceded more goals in games with at least one formation change compared to games without a formation change (*p* = 0.02; ES = 0.46; U = 49; Z = −2.36). Although the ES were mainly trivial and small they reveal more detailed information (see Supplementary Table S3). In season 1, games with a formation change were associated with fewer opposing chances and opposing scoring zone entries (ES = 0.26). In Season 2, games with a formation change had more own scoring zone entries and opponent goals and fewer own and opponent chances than games without a formation change (mean ES = 0.31). In season 3, the team created more scoring zone entries in games with formation change than in games without formation change (ES = 0.29).

The sign-tests (see Figures 3–6) revealed that the analyzed team allowed fewer opposing scoring zone entries in the 10-min-post formation change period compared to the 10-min-pre formation change period in season 1 (*p* = 0.02; ES = 1.29; positive spread = 7; negative spread = 0; and tie = 2). Further, the analyzed team created more chances in the 10-min-post formation change period compared to the 10-min-pre formation change period in *all seasons* (*p* = 0.03; ES = 0.54; positive spread = 10; negative spread = 24; and tie = 14). Subsequently, the results regarding ES reveal more detailed information (see Supplementary Table S4). Over all three seasons, the analyzed team created more goals, chances, and scoring zone entries in the 10-min-post formation change period compared to the 10-min-pre formation change period (mean ES = 0.52). Furthermore, the analyzed team prevented more opposing goals, chances, and scoring zone entries in the 10-min-post formation change period compared to the 10-min-pre formation change period (mean ES = 0.28).

## Discussion

The current study aimed to examine the effects of in-game changes in the tactical formation on goals, chances, and scoring zone entries of one team in the German Bundesliga and to analyze potential coach-specific differences regarding these effects. Generally, over all three investigated seasons, the in-game changes of tactical formation led to an improvement in the match performance of the analyzed team. In season 1, the positive effects of the in-game formation changes were the most pronounced. Therefore, the magnitude of the influence of in-game formation changes on the match performance was dependent on the season and, hence, on the coach. While the coaches in seasons 1 and 2 changed the formation when their team performed poorly, the coach in season 3 used tactical formation changes regardless of the performance of his team.

### Effects of In-Game Formation Changes

The first objective of the study was to investigate whether in-game formation changes impacted match performance. Subsequently, comparing the 10-min-pre and 10-min-post formation change periods, the changes in tactical formation had a medium positive effect on every key performance variable of attacking play (see Figures 3–5; mean ES = 0.40). All seasons combined, the variable which was affected the most by the in-game formation changes were chances of the own team (mean ES = 0.65). In conclusion, these findings suggest that in-game changes of the tactical formation helped to increase the match performance of the analyzed team in the period after the formation change. A change in the formation inevitably leads to a new tactical orientation of the team. Therefore, the opposing team is presented with new defensive and offensive tasks. Since the opponent is impaired by this change of the game, the formation change can then lead to an improvement in the offensive and defensive performance of the own team. However, since this is the first study on the effect of in-game formation changes, the results should be viewed with caution.

The improved performance after the formation change leads to an increase in own chances and a decrease in opposing chances. Lepschy et al. (2020) revealed that one critical factor determining the success in the investigated German Bundesliga is the number of shots. Regarding the present study, the key performance variable chances include shots. Summarizing, considering the results of Lepschy et al., reducing the opponent’s chances, and increasing the own team’s chances leads to a higher probability of success. Since the investigated in-game formation changes lead to this phenomenon, it is reasonable to conclude that the investigated formation changes increased the probability of success.

### Differences Between the Coaches

The second study aim, to analyze the differences between the coaches, will be addressed in the following. Subsequently, every single season will be discussed individually.

First, the coach in season 1 was able to contribute substantially to the improvement of the performance by applying in-game formation changes. The analyzed team could increase the number of goals, chances, and scoring zone entries in the 10 min after the formation change (mean ES = 0.88). Similarly, the formation changes led to fewer chances and scoring zone entries of the opposing team in the 10 min after the change (mean ES = 0.81). Moreover, the opposing team created fewer chances and scoring zone entries in games with a formation change underlining the improvement in match performance with an in-game formation change (mean ES = 0.26). Therefore, the in-game formation changes increased the match performance of the analyzed team. Previous studies revealed that different tactical formations can lead to varying offensive and defensive tactical performances (Memmert et al., 2019; Low et al., 2021). Based on those findings, if a coach wants to influence the unsatisfactory performance of his players, the change of the tactical formation is one possible tool to influence the match performance. Concluding, the formation changes of coach 1 can be valued as suitable and very effective in consideration of the respective game situation.

Second, the in-game formation changes in season 2 reveal a smaller effect on the performance of the analyzed team. On the one hand, the team scored more goals, created more chances, and conceded fewer goals in the 10 min after the formation change compared to the 10 min before (mean ES = 0.42), indicating a positive influence on performance. Furthermore, the team created more scoring zone entries and prevented more opposing chances in games with a formation change (mean ES = 0.29). On the other hand, the own scoring zone entries, opposing scoring zone entries, and opposing chances stayed rather unaffected in the 10 min after the formation change (mean ES = 0.09). Moreover, the team created fewer chances and conceded more opposing goals in games with a formation change (mean ES = 0.34). Concluding, the formation changes of the coach in season 2 improved the match performance of the analyzed team concerning opposition goals, own goals, and own chances. In contrast to season 1, the changes in tactical formation were overall less effective.

Third, in season 3, the effects of in-game changes of formation on performance were further diminished. Formation changes in this season did not affect the parameters’ own goals, opposing scoring zone entries, opposing chances, and opposing goals in the 10-min post formation change (mean ES = 0.20). Only own scoring zone entries and own chances indicate a positive alternation in the 10 min after the formation change (mean ES = 0.48). One potential conclusion could be that the coach in season 3 was focused more on the own offensive performance. Overall, the effects of in-game formation changes on the overall performance in season 3 were small. The behavior regarding scenarios leading to formation changes differed between coaches which can partly explain the differing effectiveness of formation changes between coaches. These aspects will be further discussed in the following.

### Scenarios Leading to a Formation Change

In team sports, coaches are a crucial factor in influencing player interaction during the game (Keatlholetswe and Malete, 2019). Nevertheless, coaching decisions regarding tactical formation in soccer have not yet been studied. In contrast, one investigation focusing on handball revealed that the situations in which coaches change the tactical formation during running games differed (Debanne and Laffaye, 2015). The results of this study reveal that the motivation of coaches to change a formation is influenced by different scenarios (e.g., lead) that occur in the game. Consequently, in the present study, different scenarios that motivated the respective coach to make an in-game formation change will be addressed in the following.

First, the coach in season 1 preferred to change his team formation in games where his team was less successful (∅ points in games without formation change: 1.32 ± 1.35; ∅ points in games with formation change: 1.11 ± 1.17). Another finding supporting this assumption is that the team scored fewer goals, created fewer chances, and realized fewer scoring zone entries in the 10 min before the formation change (goals = 0.00 ± 0.00; chances = 0.56 ± 0.73; and scoring zone entries = 2.22 ± 1.56) compared to the average 10 min in games with and without a formation change [(with formation change: goals = 0.17 ± 0.13; chances = 0.89 ± 0.38; scoring zone entries = 3.10 ± 0.89), (without formation change): goals = 0.17 ± 0.15; chances = 1.00 ± 0.39; scoring zone entries = 3.22 ± 0.86]. Furthermore, the opposing team earned more chances and scoring zone entries in the 10 min before a change than average in the games with and without a formation change. Concluding, throughout the nine formation changes recorded in season 1, the coach changed formations when the team underperformed compared to the team average.

Second, the decision for an in-game formation change of the coach in season 2 seemed to be dependent mainly on the parameter opposing team goals. In the 10 min previous to the formation change (goals = 0.45 ± 0.52), the opposing team scored more goals than in average 10 min in games with and without formation change ([with formation change: goals = 0.30 ± 0.21], [without formation change: goals = 0.13 ± 0.11]). Moreover, the team conceded more goals in games with a formation change compared to games without a formation change (ES = 0.46). Concluding, the coach changed the tactical formation in games where his team conceded more goals and in situations when the opposing team scored.

Third, and in contrast to the other seasons, the unclear results in season 3 do not allow a conclusion on a trigger scenario. Season 3 revealed by far the largest number of in-game formation changes (=28). Therefore, one possible explanation could be that the coach in season 3 used in-game changes of tactical formation as a tactical rationale and the effects were blurred due to the high number of formation changes. In contrast, the coaches in seasons 1 and 2 did change the tactical formation when the team showed a bad performance. However, referring to the high point averages per game (see Supplementary Table S3), the in-game decisions of the coach (changing or not changing the formation) in season 3 can still be valued as suitable. In summary, it can be said that the decision to change the formation is highly dependent on the coach and that there are interindividual differences. However, the two coaches in seasons 1 and 2 changed formation mainly when the team performed poorly, which might partially explain the higher effectiveness of formation changes during these two seasons.

In the following, the limitations of the study will be addressed. In the current investigation, the tactical formation of the opponent was not considered. As the 20 outfield players interact with each other during the game, the opposing team’s tactical formation can impact the match performance (Carling, 2011). In addition, science has already proven that the final result and the goal difference of a match have an influence on match performance (Lupo and Tessitore, 2016). However, the present study did not include the current score and final result of the investigated matches in the evaluation of the results, but only reported them in Supplementary Table S6. Science furthermore, it is necessary to address that this study only investigated the effects of in-game formation changes regarding one single team. Therefore, the generalization of the findings and conclusions to other coaches, teams, and leagues is hardly possible (Rampinini et al., 2007; Dellal et al., 2011). Moreover, because in-game formation changes are rare and we divided the results by season the sample sizes of formation changes were small. However, three full seasons of a professional soccer team were analyzed in this study. As mentioned above, the investigated team reached European competitions in two of the three analyzed seasons. The transfer toward teams with players that do not have a comparable performance quality as the players in the current study has to be questioned (Aquino et al., 2017). With the above-mentioned facts (e.g., small sample size) and the additional information that only non-parametric tests were calculated, it can be assumed that the results presented in this study are very conservative.

In contrast to the above-mentioned limitations, the present study also possesses significant strengths. First of all, the current approach is the first to evaluate the effect of in-game formation changes in soccer. Moreover, a key strength is that the tactical formations and changes in formation were observed independently by two experienced video analysts and results of both raters were reviewed until consensus was reached. Moreover, the reliability of the investigated key performance variables was checked to substantiate the significance of the results. Furthermore, the current study analyzed a professional soccer team that played on the highest level in national (i.e., Bundesliga) and international (i.e., Champions League and Europa League) competitions during the study period.

Fruitful avenues for future investigations could be to investigate the effects of in-game formation changes in other leagues and for other teams. Furthermore, addressing the opposing formation would generate additional added value to the results. Moreover, a future study could also consider longer periods after the formation change to investigate the long-term effects of the in-game changes. Furthermore, future studies could investigate other factors that potentially lead to an in-game formation change (e.g., substitutions). In addition, it is desirable to investigate a team with the same coach over a longer time. Hence, the sample sizes of in-game formation changes should get larger and, therefore, the results get more robust. Moreover, qualitative analysis (e.g., interviewing coaches) could help to put the results in a broader context. Therefore, the initial motivation of coaches to change the formation could be revealed. In addition, the psychological effects of changing the tactical formation could be studied in the future.

## Conclusion

The results of this study provide novel information about the effects of in-game formation changes in professional soccer (German Bundesliga). In-game formation changes were recorded for 43% of the games studied. Formation changes were used by different coaches for different purposes and with varying degrees of success. Across all three investigated seasons, the in-game formation changes helped the team to turn an average or below-average performance into better performance during the 10 min after the formation change. Further, the comparison between the investigated seasons indicates that the effect of the respective formation changes was dependent on the responsible coach. Different trigger scenarios were revealed that led the coaches to the in-game formation changes.

The results of the present study underpin the enormous importance of in-game decision-making of coaches. Additionally, the results reinforce the importance of coaches and their individual qualities.

## Data Availability Statement

The data that support the findings of this study are available from Wyscout on request. Restrictions apply to the availability of these data, which were used under license for this study and, therefore, are not freely available. Requests to access the datasets should be directed to www.hudl.com/support/contact.

## Ethics Statement

The studies involving human participants were reviewed and approved by Human and Business Sciences Institute, Saarland University, Germany, identification number: 22-02, 10 January 2022. Written informed consent for participation was not required for this study in accordance with the national legislation and the institutional requirements.

## Author Contributions

LeoF, SA, and TG: conceptualization. LeoF and LeaF: investigation. LeoF and SA: methodology. HW, DJ, SA, and AW: supervision. LeoF and TG: validation. LeoF, SA, LeaF, HW, DJ, AW, and TG: writing. All authors contributed to the article and approved the submitted version.

## Conflict of Interest

SA was employed by company TSG ResearchLab gGmbH.

The remaining authors declare that the research was conducted in the absence of any commercial or financial relationships that could be construed as a potential conflict of interest.

## Publisher’s Note

All claims expressed in this article are solely those of the authors and do not necessarily represent those of their affiliated organizations, or those of the publisher, the editors and the reviewers. Any product that may be evaluated in this article, or claim that may be made by its manufacturer, is not guaranteed or endorsed by the publisher.

## Supplementary Material

Supplementary Table S1 Results of inter-rater reliability for the key performance variables goals, chances, and scoring zone entries.

Supplementary Table S2 Kolmogorov-Smirnov-tests.

Supplementary Table S3 Comparison of games with and games without in-game formation change.

Supplementary Table S4 Comparison of 10-min-pre and 10-min-post in-game formation changes.

Supplementary Table S5 Descriptive information about the seasons 1-3. Single values (means± SD, where applicable).

Supplementary Table S6 Descriptive information about the in-game formation changes.
