# Supplementary material for: How Coaches Can Improve Their Teams’ Match Performance—The Influence of In-Game Changes of Tactical Formation in Professional Soccer
Source: Front Psychol. 2022 Jun 9;13:914915. doi: 10.3389/fpsyg.2022.914915 (PMC9218789; doi:10.3389/fpsyg.2022.914915)
Supplement: Supplementary Table S1 — Results of inter-rater reliability for the key performance variables goals, chances, and scoring zone entries. [file Table_1.DOCX]

**S1 Table.** Results for inter-rater reliability for the key performance variables goals, chances, and last plane entries.

|  | **Cohen‘s kappa** | **p-value** |
| --- | --- | --- |
| goals – own team | 1.00 | 0.04 |
| goals – opposing team | 1.00 | 0.08 |
| chances – own team | 1.00 | <0.01 |
| chances – opposing team | 0.62 | 0.03 |
| last plane – own team | 1.00 | <0.01 |
| last plane – opposing team | 1.00 | <0.01 |
