# Supplementary material for: How Coaches Can Improve Their Teams’ Match Performance—The Influence of In-Game Changes of Tactical Formation in Professional Soccer
Source: Front Psychol. 2022 Jun 9;13:914915. doi: 10.3389/fpsyg.2022.914915 (PMC9218789; doi:10.3389/fpsyg.2022.914915)
Supplement: Supplementary Table S2 — Kolmogorov-Smirnov-tests. [file Table_2.DOCX]

**S2 Table.** Kolmogorov-Smirnov-tests.

|  | **p-value**  **(all games)** |
| --- | --- |
| **season 1** | |
| goals – own team | **>0.01** |
| goals – opposing team | 0.07 |
| chances – own team | 0.20 |
| chances – opposing team | 0.20 |
| last plane – own team | 0.20 |
| last plane – opposing team | 0.19 |
| **season 2** | |
| goals – own team | **0.02** |
| goals – opposing team | **>0.01** |
| chances – own team | 0.20 |
| chances – opposing team | 0.17 |
| last plane – own team | 0.06 |
| last plane – opposing team | 0.20 |
| **season 3** | |
| goals – own team | **>0.01** |
| goals – opposing team | **>0.01** |
| chances – own team | 0.20 |
| chances – opposing team | **0.03** |
| last plane – own team | 0.20 |
| last plane – opposing team | 0.20 |
| **all seasons** | |
| goals – own team | **>0.01** |
| goals – opposing team | 0.07 |
| chances – own team | 0.20 |
| chances – opposing team | 0.20 |
| last plane – own team | 0.20 |
| last plane – opposing team | 0.19 |
