# Supplementary material for: How Coaches Can Improve Their Teams’ Match Performance—The Influence of In-Game Changes of Tactical Formation in Professional Soccer
Source: Front Psychol. 2022 Jun 9;13:914915. doi: 10.3389/fpsyg.2022.914915 (PMC9218789; doi:10.3389/fpsyg.2022.914915)
Supplement: Supplementary Table S3 — Comparison of games with and games without in-game formation change. [file Table_3.DOCX]

**S3 Table.** Comparison of games with and games without in-game formation change.

|  | **Average 10 min. in games without formation change**  **(mean ±SD)** | **95% Confidence intervall** | **Average 10 min. in games with formation change**  **(mean ±SD)** | **95% Confidence intervall** | **U** | **Z** | **p-value** | **ES** |
| --- | --- | --- | --- | --- | --- | --- | --- | --- |
| **season 1** | | | | | | | | |
| goals – own team | 0.17 ±0.15 | 0.04-0.26 | 0.17 ±0.13 | 0.08-0.27 | 108 | -0.18 | 0.85 | 0.01 |
| goals – opposing team | 0.17 ±0.14 | 0.12-0.30 | 0.19 ±0.11 | 0.10-0.27 | 101 | -0.47 | 0.67 | 0.03 |
| chances – own team | 1.00 ±0.39 | 0.70-1.32 | 0.89 ±0.38 | 0.60-1.18 | 94 | -0.73 | 0.47 | 0.17 |
| chances – opposing team | 0.83 ±0.37 | 0.80-1.35 | 0.69 ± 0.31 | 0.45-0.93 | 86 | -1.04 | 0.32 | 0.24 |
| last plane – own team | 3.22 ±0.86 | 2.60-3.72 | 3.10 ±0.89 | 2.41-3.79 | 109 | -0.16 | 0.88 | 0.13 |
| last plane – opposing team | 2.89 ±0.95 | 2.35-3.80 | 2.72 ±0.77 | 2.13-3.31 | 103 | -0.39 | 0.70 | 0.28 |
| **season 2** | | | | | | | | |
| goals – own team | 0.15 ±0.12 | 0.03-0.24 | 0.17 ±0.11 | 0.11-0.26 | 90 | -0.46 | 0.68 | 0.05 |
| goals – opposing team | 0.13 ±0.11 | 0.03-0.24 | 0.30 ±0.21 | 0.12-0.39 | 49 | -2.36 | **0.02** | 0.46 |
| chances – own team | 1.15 ±0.53 | 0.86-1.39 | 0.99 ±0.52 | 0.65-1.45 | 80 | -0.91 | 0.37 | 0.22 |
| chances – opposing team | 1.16 ±0.43 | 0.64-1.48 | 0.96 ±0.43 | 0.61-1.12 | 79 | -0.95 | 0.35 | 0.31 |
| last plane – own team | 2.94 ±0.87 | 2.38-3.20 | 3.21 ±1.26 | 2.54-4.30 | 83 | -0.77 | 0.45 | 0.27 |
| last plane – opposing team | 3.86 ±1.04 | 2.86-4.28 | 3.48 ±1.27 | 2.39-4.13 | 82 | -0.79 | 0.45 | 0.19 |
| **season 3** | | | | | | | | |
| goals – own team | 0.21 ±0.12 | 0.11-0.28 | 0.24 ±0.16 | 0.11-0.36 | 122 | -0.39 | 0.71 | 0.06 |
| goals – opposing team | 0.15 ±0.10 | 0.10-0.25 | 0.18 ±0.14 | 0.06-0.23 | 113 | -0.72 | 0.51 | 0.10 |
| chances – own team | 1.28 ±0.55 | 0.85-1.62 | 1.15 ±0.46 | 0.85-1.57 | 116 | -0.58 | 0.58 | 0.19 |
| chances – opposing team | 0.87 ±0.39 | 0.63-1.20 | 0.84 ±0.38 | 0.49-1.19 | 126 | -0.24 | 0.82 | 0.04 |
| last plane – own team | 3.41 ±0.79 | 2.78-4.06 | 3.70 ±1.10 | 3.15-4.97 | 118 | -0.51 | 0.63 | 0.29 |
| last plane – opposing team | 2.84 ±0.69 | 2.24-3.39 | 2.78 ±1.17 | 1.74-3.52 | 124 | -0.29 | 0.79 | 0.06 |
| **all seasons** | | | | | | | | |
| goals – own team | 0.17 ±0.13 | 0.14-0.21 | 0.21 ±0.14 | 0.16-0.25 | 1003 | -1.29 | 0.20 | 0.09 |
| goals – opposing team | 0.15 ±0.12 | 0.12-0.20 | 0.21 ±0.16 | 0.16-0.26 | 909 | -1.95 | 0.05 | 0.16 |
| chances – own team | 1.11 ±0.48 | 0.94-1.20 | 1.05 ±0.46 | 0.91-1.20 | 1045 | -0.89 | 0.37 | 0.08 |
| chances – opposing team | 0.96 ±0.84 | 0.81-1.04 | 0.84 ±0.38 | 0.72-0.96 | 994 | -1.27 | 0.21 | 0.19 |
| last plane – own team | 3.16 ±0.85 | 2.91-3.39 | 3.45 ±1.11 | 3.10-3.80 | 990 | -1.29 | 0.20 | 0.29 |
| last plane – opposing team | 3.20 ±0.99 | 2.89-3.51 | 2.93 ±1.14 | 2.57-3.30 | 992 | -1.28 | 0.20 | 0.26 |
