# Supplementary material for: How Coaches Can Improve Their Teams’ Match Performance—The Influence of In-Game Changes of Tactical Formation in Professional Soccer
Source: Front Psychol. 2022 Jun 9;13:914915. doi: 10.3389/fpsyg.2022.914915 (PMC9218789; doi:10.3389/fpsyg.2022.914915)
Supplement: Supplementary Table S4 — Comparison of 10-min-pre and 10-min-post in-game formation changes. [file Table_4.DOCX]

**S4 Table.** Comparison of 10-min-pre and 10-min-post in-game formation changes.

|  | **Average 10 min. pre in game formation change**  **(mean ±SD)** | **95% Confidence intervall** | **Average 10 min. post in game formation change**  **(mean ±SD)** | **95% Confidence intervall** | **positive spread** | **negative spread** | **tie** | **p-value** | **ES** |
| --- | --- | --- | --- | --- | --- | --- | --- | --- | --- |
| **season 1** | | | | | | | | | |
| goals – own team | 0.00 ±0.00 | 0.00-0.00 | 0.33 ±0.50 | -0.05-0.72 | 0 | 3 | 6 | 0.25 | 0.67 |
| goals – opposing team | 0.11 ±0.33 | -0.15-0.37 | 0.11 ±0.33 | -0.15-0.37 | 1 | 1 | 7 | >0.99 | 0.00 |
| chances – own team | 0.56 ±0.73 | 0.00-1.11 | 1.56 ±1.51 | 0.40-2.72 | 1 | 5 | 3 | 0.22 | 0.95 |
| chances – opposing team | 1.33 ±0.87 | 0.67-2.00 | 1.00 ±1.12 | 0.14-1.86 | 4 | 1 | 4 | 0.38 | 0.33 |
| last plane – own team | 2.22 ±1.56 | 1.02-3.42 | 3.56 ±1.88 | 2.11-5.00 | 2 | 5 | 2 | 0.45 | 1.02 |
| last plane – opposing team | 4.22 ±2.05 | 2.65-5.80 | 2.44 ±1.74 | 1.11-3.78 | 7 | 0 | 2 | **0.02** | 1.29 |
| **season 2** | | | | | | | | | |
| goals – own team | 0.18 ±0.40 | -0.15-0.37 | 0.36 ±0.50 | -0.05-0.72 | 2 | 4 | 5 | 0.69 | 0.27 |
| goals – opposing team | 0.45 ±0.52 | 0.15-0.96 | 0.18 ±0.40 | -0.15-0.37 | 4 | 1 | 6 | 0.38 | 0.40 |
| chances – own team | 1.18 ±1.08 | 0.47-2.19 | 1.91 ±1.81 | 0.53-3.24 | 2 | 4 | 5 | 0.69 | 0.60 |
| chances – opposing team | 0.64 ±0.92 | -0.10-1.44 | 0.64 ±0.81 | 0.00-1.11 | 2 | 2 | 7 | >0.99 | 0.00 |
| last plane – own team | 3.73 ±1.56 | 2.46-5.10 | 3.91 ±2.21 | 1.98-5.57 | 4 | 5 | 2 | >0.99 | 0.13 |
| last plane – opposing team | 2.81 ±1.40 | 1.84-3.94 | 2.64 ±2.50 | 1.17-5.05 | 5 | 5 | 1 | >0.99 | 0.13 |
| **season 3** | | | | | | | | | |
| goals – own team | 0.25 ±0.44 | -0.15-0.37 | 0.29 ±0.53 | -0.12-0.56 | 5 | 6 | 17 | >0.99 | 0.05 |
| goals – opposing team | 0.21 ±0.42 | -0.15-0.37 | 0.11 ±0.31 | -0.15-0.37 | 6 | 3 | 19 | 0.51 | 0.18 |
| chances – own team | 0.96 ±1.14 | -0.06-1.62 | 1.39 ±1.26 | 0.77-3.01 | 7 | 15 | 6 | 0.13 | 0.39 |
| chances – opposing team | 0.86 ±0.93 | 0.00-1.11 | 0.82 ±0.67 | 0.12-1.21 | 11 | 10 | 7 | >0.99 | 0.04 |
| last plane – own team | 3.64 ±1.93 | 3.11-5.78 | 4.50 ±2.60 | 3.23-7.66 | 8 | 14 | 6 | 0.29 | 0.57 |
| last plane – opposing team | 2.43 ±1.75 | 0.85-3.15 | 2.57 ±1.83 | 1.53-5.14 | 9 | 13 | 6 | 0.52 | 0.11 |
| **all seasons** | | | | | | | | | |
| goals – own team | 0.19 ±0.39 | 0.03-0.26 | 0.31 ±0.51 | 0.15-0.48 | 7 | 13 | 28 | 0.26 | 0.18 |
| goals – opposing team | 0.25 ±0.44 | 0.11-0.38 | 0.13 ±0.33 | 0.02-0.23 | 11 | 5 | 32 | 0.21 | 0.19 |
| chances – own team | 0.94 ±1.06 | 0.68-1,37 | 1.54 ±1.43 | 1.21-2.15 | 10 | 24 | 14 | **0.03** | 0.54 |
| chances – opposing team | 0.90 ±0.93 | 0.57-1.09 | 0.82 ±0.79 | 0.52-1.04 | 17 | 13 | 18 | 0.58 | 0.09 |
| last plane – own team | 3.40 ±1.84 | 3.08-4.24 | 4.19 ±2.39 | 3.65-5.18 | 14 | 24 | 10 | 0.14 | 0.54 |
| last plane – opposing team | 2.85 ±1.83 | 2.23-3.33 | 2.56 ±1.95 | 1.86-3.17 | 21 | 18 | 9 | 0.75 | 0.19 |
