# Supplementary material for: How Coaches Can Improve Their Teams’ Match Performance—The Influence of In-Game Changes of Tactical Formation in Professional Soccer
Source: Front Psychol. 2022 Jun 9;13:914915. doi: 10.3389/fpsyg.2022.914915 (PMC9218789; doi:10.3389/fpsyg.2022.914915)
Supplement: Supplementary Table S5 — Descriptive information about the seasons 1-3. Single values (means± SD, where applicable). [file Table_5.DOCX]

**S5 Table.** Descriptive information about the seasons 1-3. Single values (means ±SD, where applicable).

|  | **season 1** | **season 2** | **season 3** |
| --- | --- | --- | --- |
| **Information about the seasons** | | | |
| Games incuded | 34 | 30 | 34 |
| games without formation change | 25 | 20 | 12 |
| games with formation change | 9 | 10 | 22 |
| ø points (games without formation change) | 1.32 ±1.35 | 1.55 ±1.28 | 1.58 ±1.08 |
| ø points (games with formation change) | 1.11 ±1.17 | 1.20 ±1.55 | 1.45 ±1.37 |
| **formation changes in detail:** | | | |
| formation changes total | 9 | 11 | 28 |
| ø minute of play of formation change | 64.11 ±15.57 | 55.82 ±13.20 | 55.46 ±17.45 |
| ø game day of formation change | 20.00 ±10.56 | 10.73 ±6.37 | 20.59 ±10.64 |
| ø points at moment of formation change | 1.22 ±1.39 | 0.45 ±0.93 | 1.61 ±1.37 |
| combined changes in offensive & defensive formation | 7 | 5 | 16 |
| changes only in defensive formation | 1 | 2 | 8 |
| changes only in offensive formation | 1 | 4 | 4 |
