# Supplementary material for: How Coaches Can Improve Their Teams’ Match Performance—The Influence of In-Game Changes of Tactical Formation in Professional Soccer
Source: Front Psychol. 2022 Jun 9;13:914915. doi: 10.3389/fpsyg.2022.914915 (PMC9218789; doi:10.3389/fpsyg.2022.914915)
Supplement: Supplementary Table S6 — Descriptive information about the in-game formation changes. [file Table_6.DOCX]

**S6 Table.** Descriptive information about the in-game formation changes.

| **matchday** | **defensive formation [before change]** | **defensive formation**  **[after change]** | **offensive formation [before change]** | **offensive formation**  **[after change]** | **minute** | **opposition quality**  **[table position end of the season]** | **current score**  **[own team:opposing team]** | **end result**  **[own team:opposing team]** | **home/away match** |
| --- | --- | --- | --- | --- | --- | --- | --- | --- | --- |
| **season 1** | | | | | | | | | |
| 5 | 5-2-3 | 5-3-2 | 4-2-2-2 | 4-2-2-2 | 41 | 17 | 1:1 | 1:1 | away |
| 8 | 3-4-2-1 | 4-4-2 diamond | 3-4-2-1 | 4-3-3 | 45 | 9 | 1:2 | 3:3 | home |
| 10 | 4-4-2 diamond | 4-2-3-1 | 4-4-2 diamond | 4-2-3-1 | 69 | 13 | 3:1 | 3:1 | home |
| 14 | 4-2-3-1 | 4-2-3-1 | 4-2-3-1 | 3-4-3 diamond | 49 | 10 | 0:3 | 1:3 | home |
| 26 | 5-2-3 | 4-4-2 diamond | 3-4-2-1 | 4-4-2 diamond | 76 | 12 | 1:2 | 1:2 | home |
| 27 | 5-3-2 | 4-2-3-1 | 3-4-1-2 | 4-2-3-1 | 75 | 13 | 0:2 | 1:2 | away |
| 29 | 4-2-3-1 | 5-3-2 | 4-2-3-1 | 3-1-4-2 | 62 | 2 | 0:0 | 0:0 | away |
| 30 | 4-2-3-1/4-4-2 | 5-3-2 | 4-2-3-1 | 3-1-4-2 | 83 | 8 | 3:2 | 3:2 | home |
| 31 | 4-2-3-1/4-4-2 | 5-3-2 | 4-2-3-1 | 3-1-4-2 | 77 | 10 | 1:0 | 1:1 | away |
| **season 2** | | | | | | | | | |
| 2 | 5-4-1 | 5-4-1 | 3-4-3 | 3-1-5-1 | 46 | 16 | 0:1 | 3:2 | home |
| 4 | 3-4-3 | 4-3-3 | 3-4-1-2 | 4-3-3 | 46 | 8 | 0:2 | 0:3 | home |
| 4 | 4-3-3 | 3-5-2 | 4-3-3 | 3-5-2 | 70 | 8 | 0:3 | 0:3 | home |
| 7 | 4-1-4-1 | 5-4-1 | 3-4-2 | 3-4-2 | 82 | 1 | 2:1 | 2:1 | away |
| 8 | 3-4-3 | 3-5-2 | 4-1-3-2 | 3-5-2 | 45 | 12 | 0:0 | 2:0 | home |
| 11 | 3-5-2 | 3-4-3 | 3-4-2-1 | 3-4-2-1 | 46 | 14 | 0:1 | 2:1 | away |
| 12 | 3-4-3 | 3-4-3 | 3-4-2-1/3-1-5-1 | 3-2-3-2 | 55 | 13 | 0:2 | 1:5 | home |
| 15 | 4-2-3-1 | 4-2-3-1 | 4-2-2-2/3-1-5-1 | 3-5-2 | 61 | 15 | 1:3 | 2:4 | home |
| 15 | 4-2-3-1 | 4-2-3-1 | 3-5-2 | 4-1-4-1 | 71 | 15 | 1:3 | 2:4 | home |
| 18 | 4-3-3 | 3-5-2 | 4-3-3/3-1-5-1 | 3-5-2 | 46 | 9 | 0:1 | 1:2 | home |
| 22 | 3-5-2 | 4-2-3-1 | 3-1-5-1 | 4-2-3-1 | 46 | 7 | 1:1 | 2:3 | home |
| **season 3** | | | | | | | | | |
| 3 | 4-3-2-1 | 3-3-2-2 | 3-1-5-1 | 4-1-3-2 | 41 | 10 | 0:0 | 1:2 | away |
| 3 | 3-3-2-2 | 4-3-3 | 4-1-3-2 | 4-1-3-2 | 72 | 10 | 0:1 | 1:2 | away |
| 15 | 4-1-4-1 | 4-1-4-1 | 3-4-2-1 | 3-5-2 | 65 | 5 | 0:0 | 0:0 | home |
| 18 | 3-5-2 | 4-3-3 | 3-1-5-1 | 3-1-5-1 | 46 | 1 | 0:2 | 1:3 | home |
| 20 | 3-5-2 | 3-5-2 | 3-1-5-1 | 3-5-2 | 73 | 10 | 1:1 | 1:1 | home |
| 22 | 4-4-2 diamond | 3-5-2 | 3-1-3-3 | 3-1-3-3 | 46 | 17 | 2:0 | 3:0 | home |
| 24 | 3-1-5-1 | 4-2-3-1 | 3-1-5-1 | 4-2-2-2 | 38 | 7 | 0:1 | 2:3 | away |
| 24 | 4-2-3-1 | 4-4-1 | 4-2-2-2 | 4-4-1 | 67 | 7 | 2:1 | 2:3 | away |
| 25 | 3-4-1-2 | 3-5-2 | 3-5-2 | 4-3-3 | 70 | 18 | 1:1 | 2:1 | home |
| 28 | 3-5-2 | 3-5-2 | 4-2-2-2 | 3-4-3 | 62 | 15 | 2:0 | 4:0 | away |
| 28 | 3-5-2 | 5-4-1 | 3-4-3 | 3-4-3 | 46 | 15 | 1:0 | 4:0 | away |
| 30 | 3-4-1-2 | 4-2-2-2 | 3-1-5-1 | 4-2-2-2 | 56 | 14 | 2:0 | 5:2 | away |
| 30 | 4-2-2-2 | 3-4-3 | 4-2-2-2 | 3-4-3 | 71 | 14 | 3:1 | 5:2 | away |
| 31 | 4-4-2 Raute | 3-5-2 | 4-2-2-2 | 3-4-3 | 12 | 6 | 1:0 | 1:4 | home |
| 31 | 3-5-2 | 5-4-1 | 3-4-3 | 4-2-4 | 73 | 6 | 1:2 | 1:4 | home |
| 33 | 4-4-2 diamond | 3-4-3 | 3-5-2 | 3-5-2 | 46 | 8 | 0:1 | 0:1 | home |
| 33 | 3-4-3 | 3-5-2 | 3-5-2 | 3-5-2 | 60 | 8 | 0:1 | 0:1 | home |
| 2 | 5-3-2 | 5-4-1 | 4-4-2 | 4-3-3 | 46 | 13 | 0:1 | 3:1 | home |
| 6 | 4-2-3-1 | 4-2-3-1 | 3-5-2 | 4-3-3 | 78 | 3 | 0:2 | 1:2 | home |
| 7 | 5-3-2 | 4-3-3 | 3-5-2 | 4-3-3 | 66 | 7 | 0:2 | 1:2 | home |
| 9 | 4-4-2 diamond | 5-3-2 | 4-4-2 diamond | 3-3-3-1 | 29 | 16 | 0:0 | 4:0 | home |
| 10 | 5-4-1 | 5-3-2 | 3-4-3 | 3-5-2 | 60 | 4 | 3:1 | 4:1 | away |
| 11 | 4-3-3 | 5-3-2 | 4-3-3 | 3-5-2 | 72 | 15 | 1:1 | 2:1 | home |
| 12 | 4-5-1 | 5-3-2 | 3-1-4-2 | 3-1-4-2 | 16 | 11 | 2:1 | 3:3 | away |
| 19 | 4-4-2 diamond | 5-3-2 | 4-4-2 diamond | 3-5-2 | 65 | 13 | 2:1 | 4:2 | away |
| 23 | 4-4-2 diamond | 5-3-2 | 3-1-5-1 | 3-5-2 | 65 | 3 | 1:0 | 1:1 | away |
| 32 | 4-2-3-1 | 5-3-2 | 3-1-4-2 | 3-1-4-2 | 70 | 5 | 1:0 | 2:2 | away |
| 34 | 4-3-3 | 4-4-1 | 3-4-3 | 4-4-1 | 42 | 12 | 2:0 | 2:4 | away |
